# COVID-19 pandemic: perception, attitude, and practices of university students from health sector faculties

**DOI:** 10.1186/s43168-023-00177-7

**Published:** 2023-01-16

**Authors:** Basma Mohamed Osman, Shaimaa S. Abdelrheem, Ramy M. El Sabaa, Farida Kamel Yousef, Aliae A. R. Mohamed Hussein, Nermen M. Abuelkassem, Salwa A. Atlam

**Affiliations:** 1 Faculty of Nursing, Cairo University, Cairo, Egypt; 2 Public Health and Community Medicine department, Faculty of Medicine, Aswan University, Aswan, Egypt; 3 Clinical Pharmacy, Faculty of Pharmacy, Deraya University, New Minya, Egypt; 4 Department of Family and Community Health Nursing, Faculty of Nursing, Suez Canal University, Ismailia, Egypt; 5 Chest Department, Assiut University Hospitals, Assiut University, Assiut, Egypt; 6 Public Health and Community Medicine department, Faculty of Medicine, Tanta University, Tanta, Egypt

**Keywords:** Attitude, COVID-19, Healthcare, Perception, Practice, Students

## Abstract

**Background:**

Adherence to preventive control measures is influenced by perception, attitudes, and practices toward the disease prevention.

**Aim:**

To assess the perceptions, attitude, and practices of university students in three health sector faculties (Medicine, Nursing, and Pharmacy) from six Egyptian universities towards COVID-19 pandemic prevention.

**Methods:**

An electronic online survey was distributed to students of 3 faculties (Medicine, Pharmacy, and Nursing) in six Egyptian universities from March to July 2021. The questionnaire consists of the following parts: socio-demographic data of participants, student perception and attitude towards the COVID-19 pandemic and its consequences, as well as practices of preventive measures in the community.

**Results:**

The study included 1990 participants. Most respondents perceived the seriousness of the COVID-19 pandemic (88.2%). The rates of practicing precautionary measures were mostly high (approximately 90% or above). Significantly high attitude scores toward the pandemic were detected in people with quite-to-extremely concerned, very good/excellent status, aware of infection risk, willing to report positive cases and avoiding contact with them, and seeking medical advice regarding infection. The attitude to protective measures followed a similar pattern along with average health status, female students, and avoidance of contact when experiencing flu symptoms. Significantly higher practice scores were observed in those with bad health status, rural areas, perceiving the risk of infection, willing to report positive cases, and avoiding contact with positive cases.

**Conclusion:**

The effectiveness of precautionary measures should be stressed to promote positive student practices.

## Introduction

The world is currently experiencing a novel pandemic named severe acute respiratory syndrome coronavirus 2 (SARS-COV2) that causes COVID-19 disease [1]. The first cases were reported in Wuhan city, China in 2019 [2]. On the 30th of January 2020, the World Health Organization (WHO) declared the COVID-19 outbreak to be a public health emergency. On the 11th of March 2020, the WHO declared the outbreak a pandemic [3]. According to the emergency situational updates of the WHO on September 2021, the cumulative number of reported COVID-19 cases and related deaths worldwide has reached over 224 million and over 4.6 million, respectively [4].

As the search continues for an effective cure for COVID-19, preventing the virus spread is the best strategy against this pandemic through fostering positive attitudes and beliefs that enable practicing the recommended protective strategies [5] such as washing hands with soap and water or using hand sanitizers, covering the mouth and nose while coughing or sneezing, avoiding crowded places, adhering to social distancing, avoiding close contact with suspected cases [6].

In addition, healthcare workers and students attending health sector faculties are anticipated to strictly adhere to the recommended protective measures because they are at increased risk for contracting COVID-19 due to their encounters with patients [7–9].

Previous studies have shown that nursing students during epidemics are exposed to severe psychological stress and anxiety that have a serious impact on their education and psychological health [8]. For example, the interruption of education for nursing students has been unexpected. Clinical practice of nursing students in hospitals has also been discontinued, and students may have been concerned about inadequate clinical skill development. In addition, in sufficient application; skills, and uncertainty about when, where, and how compensatory training is conducted to eliminate inadequacy, can stress nursing students [10].

Although medical, pharmacy, and nursing students are not usually in direct contact with highly contagious cases, undergraduate medical and nursing students are considered in the current pandemic as the backup force during the peaks of the pandemic waves in the event of healthcare worker shortage [11]. Some countries already have involved medical and nursing students in their final years in hospitals since the start of the pandemic [12]. This imposes a duty on all medical and nursing students to protect themselves and to limit the spread of infection to others.

Risk Communication and Community Engagement (RCCE) is an essential part of a health emergency preparedness and response action plan [3]. Risk perception plays a major role in estimating the extent to which community is aware of the seriousness of this pandemic and the extent of the willingness to cooperate in the implementation of health preventive measures at the individual, local, and international levels [13].

In addition, medical, pharmacy, and nursing students in many communities are considered role models in matters of health from which reliable information could be obtained. Therefore, they can combat the myths and misconceptions surrounding the diseases and disseminate sound scientific knowledge about the pandemic and how to safeguard against infection, particularly if the students’ behavior towards the pandemic is exemplary. Therefore, the present study aimed to assess the perceptions and attitudes of Egyptian medical and nursing students towards the COVID-19 pandemic and the compliance with recommended precautionary measures.

## Methods

### Study design

A cross-sectional survey study was utilized in which data were collected following the Checklist for Reporting Results of Internet E Surveys (CHERRIES) guidelines during the period from March to July 2021.

### Study settings and participants

The study conducted through an electronic anonymous online survey distributed among students from three health sector faculties (Medicine, Pharmacy, and Nursing) in six Egyptian universities (Cairo, Suez Canal, Tanta, Deraya, Aswan, Kufr Elsheikh).

The self-administered online questionnaire was hosted in Google form and the link was distributed through official university platforms and informal student groups on social media like Facebook and WhatsApp pages. Representatives of students were also engaged in distributing the questionnaire’s link directly to their colleagues from each faculty. A non-randomized convenient sample of 1990 university medical students was included in the study**.**

### Data collection

The study questionnaire was developed based on the COVID-19 Rapid Quantitative Assessment Tool as part of the Risk Communication and Community Engagement (RCCE) Action Plan Guidance COVID-19 preparedness and response developed by the China International Famine Relief Commission publication, United Nations Children’s Fund (UNICEF) and World Health Organization (WHO) [14]. Most of the questions were in the form of “yes and no” questions except for a few questions with the third “I don’t know” option, a five-point rating scale question.

After the tool was developed, the application was tested by the EYI (Egyptian Youth Initiative) executive team at the National Population Council (NPC)/UNICEF to verify, confirm and skip patterns and follow them to guarantee the accuracy of data collection. To ensure the validity and accuracy of the data collected, the investigators settle detailed instructions regarding study objectives for all student counselors who distributed the questionnaire to the subjects in advance. The survey tool automatically verifies that all questions must be filled before submission and cannot be submitted twice.

#### The questionnaire consists of the following parts:

Socio-demographics of study participants (age, gender, university, faculty, place of residence, family income, and socioeconomic status).

##### COVID-19 infection risk perception

COVID-19 is a dangerous disease, perceived risk of infection, concerns towards COVID-19 pandemic, perceived health status, perceived COVID-19 information adequacy, and adequate information).

COVID-19 impact on students’ health and quality of life. Four items were included: how the COVID-19 pandemic affected your health, studies, education, and mental health (psychological distress due to COVID-19) and disturbed your daily activities and lifestyle.

##### Attitude towards COVID-19 prevention

A five-item attitudinal statement was used to assess the level of trust and confidence regarding the protective measures: wearing a mask, covering the nose and mouth during sneezing and coughing, social distancing, following good hygienic measures (washing hands with soap and water using disinfectant).

Participants were then asked whether maintaining these protective measures in the community could reduce the pandemic and whether or not they have confidence in the newly discovered COVID-19 vaccine. In addition, “What would they do if they had close contact with positively active case? Do they avoid contact with a positively confirmed case? In addition, if any of them experience Flu symptoms will he/she avoid contact with others. Would they seek medical advice in the case of confirmed infection? Participants were requested to mention barriers to implementing public health measures to prevent the spread of infection in the community.

### Practices toward COVID-19 prevention in the community

Five questions were asked to assess participants’ practices to protect themselves and others. “Do you use facemask when leaving home? Do you avoid crowded places to protect yourself? Do you practice social distancing to protect yourself and others? Do you use disinfectant and gel to wash your hands regularly? Do you cove your nose and mouth with tissue during sneezing and coughing? “

The questionnaire was developed in Arabic language using a simple local language and was previously tested in a pilot study with 30 participants from the six universities (other than those studied). The questionnaire reliability was confirmed by applying a reliability test using Cronbach’s alpha (0.73). To ensure the validity and accuracy of collected data, the researchers provided detailed instructions about the study objectives to all student counselors who distributed the questionnaire to subjects beforehand. The survey tool automatically verified that all questions had to be filled before submission and cannot be submitted twice.

#### Scoring system

The research team used a standard scoring system of five-point Likert rating scale items for the attitude sections (not at all, mild, moderate, considerably affected, highly affected). The five items related to the level of trust towards COVID-19 protective measures had a score ranging from 0 to 25 points. The score was calculated from the four variables representing the participants’ attitude towards the impact of the pandemic in relation to their studies, health status, mental health, and daily activities (0–15 points). A maximum total score of 45 points for the aforementioned nine variables was calculated to assess the participants’ total attitude score. For the practice score, five questions about the performance of protective measures were included and graded using a three-point Likert scale (sometimes, regularly, all the time), with a score ranging from 0 to 20 points. Coded questions with multiple correct responses and a separate variable for each valid answer to the question created for analysis. Using dichotomy sets, each variable has two possible scores, which indicate the response selection by the survey taker.

### Ethical considerations

Research ethical approval from the faculty of pharmacy, Draya University Research Ethical Committee (REC) was obtained. Study participants provided written informed consent electronically after being informed of the purpose of the study and before data collection. Those who agreed to participate in the study completed the submission process. The data has been maintained confidentially in accordance with the revised Helsinki deceleration of biomedical ethics.

#### Data processing and analysis

The data were analyzed using the Statistical Package for the Social Sciences (SPSS) 22.0 software (IBM Microsoft). For quantitative variables, the mean and standard deviation were used to express the results. Independent *t*-test and one-way ANOVA were to compare between means. For qualitative variables, frequencies were used to express nominal and ordinal variables. The adopted level of significance was *p* < 0.05

## Results

The number of students who responded to the questionnaire was 1990. Table 1 demonstrates the socio-demographic characteristics of the study participants. All of them were in the age group from 18 to 24 years, 68.3% were female, and 58% were from urban communities. Less than half of them (44.5%) have an average family income (enough and saving), and 40.5% of average social status.

**Table 1:** COVID-19 pandemic and characteristics of the study participants (*n*=1990)

Variables	Male	Female	Total	***p***-value
	***N***=633 (31.7%)	***N***= 1360 (68.3 %)	***N***= 1990 (100%)	
**Residences**				
Urban	399 (63.3)	756 (55.6)	1155 (58.0)	0.001*
Rural	231 (36.7)	604 (44.4)	835 (42.0)	
**Family income**				
Not enough	79 (12.5)	262 (19.3)	341 (17.1)	0.001 *
Enough/not saving	248 (39.4)	516 (37.9)	764 (38.4)	
Enough/saving	303 (48.1)	582 (42.8)	885 (44.5)	
**Social status**				
Low	105 (16.7)	152 (11.2)	257 (12.9)	0.003*
Average	289 (45.9)	676 (49.7)	965 (48.5)	
High	236 (37.5)	532 (39.1)	768 (38.6)	
**COIVID-19 is a dangerous disease**				
Yes	566 (89.9)	1190 (87.5)	1756 (88.2)	0.281
Don’t know	46 (7.3)	128 (9.4)	174 (8.7)	
No	18 (2.9)	42 (3.1)	60 (3.0)	
**Perceived risk of COVID-19 infection**				
Yes	428 (67.9)	714 (52.5)	1142 (57.4)	0.001*
No	202 (32.1)	646 (47.5)	848 (42.6)	
**Concerns towards COVID-19 pandemic**				
Not concerned	65 (10.3)	108 (7.9)	173 (8.7)	0.020*
Well concerned	185 (29.4)	355 (26.1)	540 (27.1)	
Quite concerned	288 (45.7)	640 (47.1)	928 (46.6)	
Extremely concerned	92 (14.6)	257 (18.9)	349 (17.5)	
**Perceived health status**				
Bad/fair	428 (67.9)	714 (52.5)	1142 (57.4)	0.001*
Average/good	109 (17.3)	493 (36.3)	602 (30.3)	
Very good/excellent	93 (14.8)	153 (11.3)	246 (12.4)	
**Getting sufficient information about COVID-19**				
Yes	443 (70.3)	912 (67.1)	1355 (68.1)	0.147
No	187 (29.7)	448 (32.9)	635 (31.9)	
**What would you do if you had close contact with confirmed cases?**				
Proactively report	542 (86.0)	1008 (74.1)	1550 (77.9)	0.001*
I don’t know	88 (14.0)	352 (25.9)	440 (22.1)	
**Do you avoid contact with a positively confirmed case?**				
Yes	613 (97.3)	1345 (98.9)	1958 (98.4)	0.050
No	17 (2.7)	15 (1.1)	32 (1.6)	
**If you have Flu symptoms, do you avoid contact with others**				
Yes	528 (83.8)	1257 (92.4)	1785 (89.7)	0.001*
No	102 (16.2 )	103 (7.6)	205 (10.3)	
**Would you seek medical advice if you experience any COVID-19 symptoms**				
Yes	612 (97.1)	1318 (96.9)	1930 (97.0)	0.779
No	18 (2.9)	42 (3.1)	60 (3.0 )	
**Do you think that maintaining protective measures will reduce infection**				
Yes	388 (61.6)	803 (59.0)	1191 (59.8)	0.282
No	242 (38.4)	557 (41.0 )	799 (40.2)	
**Do you think that the newly discovered vaccine has many side effects**				
Yes	280 (44.4)	651 (47.9)	931 (46.8)	0.155
No	350 (55.6 )	709 (52.1)	1059 (53.2)	

*Statistically significant at *p*<0.05

The majority (88.2%) considered COVID-19 to be a serious disease. Nearly half of them (57.4%) consider themselves to be at risk of infection. Almost 64.1% are quite and extremely concerned about the pandemic. Only one-third (30.3%) perceived their health status as average/good. Most of them (68.1%) were getting sufficient information about COVID-19. More than 75% would proactively report to the authority if they had been in close contact with a confirmed case and the majority (98.4%), were cautious to avoid close contact with any positively active case. Almost 90% will avoid contact with others if they experience any flu symptoms and 97% will seek medical advice if they had COVID-19 symptoms. More than half (59.8%) of them believe that maintaining protective measures in the community would reduce the spread of infection in the community. Nearly half (46.8%) believe that the COVID-19 vaccine has many side effects. The differences between the male and female participants are presented in Table 1.

Table 2 shows the effect of the pandemic on the students’ mental health and psychological disorders, health status, study achievement/educational performance, and their daily activities. During the past three months, as perceived by students , statistically significant differences were detected between male and female students regarding their psychological distress and health status towards the pandemic (*p* = 0.001 and 0.019, respectively).

**Table 2:** Participant’s attitudes and practices toward the COVID-19 pandemic by gender (*n*=1990)

Variables	Male	Female	Total	***p***-value
	***N***= 633 (31.7%)	***N***=1360 (68.3 %)	***N***= 1990 (100%)	
**How disturbed have you been in the past 3 months regarding your**				
**Mental Health**				
Mild	78 (12.4)	120 (8.8)	198 (9.9)	0.001*
Moderate	203 (32.2)	373 (27.4)	576 (28.9)	
Severe	349 (55.4)	867 (63.8)	1216 (61.1)	
**Health status**				
Mild	168 (26.7)	385 (28.3)	553 (27.8)	0.019*
Moderate	195 (31.0)	486 (35.7)	681 (34.2)	
Severe	267 (42.4)	489 (36.0)	756 (38.0)	
**Studies achievement**				
Mild	70 (11.1)	113 (8.3)	183 (9.2)	0.129
Moderate	248 (39.4)	559 (41.1)	807 (40.6)	
Severe	312 (49.5)	688 (50.6)	1000 (50.3)	
**Daily activities**				
Mild	38 (6.0)	85 (6.3)	123 (6.2)	0.956
Moderate	72 (11.4)	160 (11.8)	232 (11.7)	
Severe	520 (82.5)	1115 (82.0)	1635 (82.2)	
**Trusting the protective measures (Level of confidence)**				
**Wearing the mask**				
Low	24 (3.8)	28 (2.1)	52 (2.6)	0.030*
Moderate	177 (28.1)	356 (26.2)	533 (26.8)	
High	429 (68.1)	976 (71.8)	1407 (70.46)	
**Covering nose and mouth during sneezing and coughing**				
Low	17 (2.7)	9 (0.7)	26 (1.3)	0.001*
Moderate	163 (25.9)	294 (21.6)	457 (23.0)	
High	450 (71.4)	1057 (77.1)	1507 (75.7 )	
**Social distancing**				
Low	23 (3.7)	18 (1.3)	41 (2.1)	0.003*
Moderate	152 (24.1)	332 (24.4)	484 (24.3)	
High	455 (72.2)	1010 (74.3)	1465 (73.6)	
**Washing hands with soap and water**				
Low	15 (2.4)	9 (0.7)	24 (1.2)	0.001*
Moderate	162 (25.7)	302 (22.2)	464 (23.3)	
High	453 (71.9)	1049 (77.1)	1502 (75.5)	
**Practices of precautionary actions in the community**				
**Do you use a facemask when leaving home?**				
Sometimes	105 (16.7)	152 (11.2)	257 (12.9)	0.003*
Regularly	289 (45.9)	676 (49.7)	965 (48.5)	
All the time	236 (37.5)	532 (39.1)	768 (38.6)	
**Do you avoid crowded places to protect yourself from COVID-19?**				
Sometimes	79 (12.5)	268 (19.3)	341 (17.1)	0.001*
Regularly	248 (39.4)	516 (37.9)	764 (38.4)	
All the time	303 (48.1)	582 (42.8)	885 (44.5)	
**Do you practice social distancing to protect yourself?**				
Sometimes	162 (25.7)	363 (26.7)	525 (26.4)	0.003*
Regularly	215 (34.1)	367 (27.0)	582 (29.2)	
All the time	253 (40.2)	630 (46.3)	883 (44.4)	
**Do you use disinfectant or hand gel to wash your hands to protect yourself?**				
Sometimes	113 (17.9)	160 (11.8)	273 (13.7)	0.001*
Regularly	191 (30.3)	379 (27.9)	570 (28.9)	
All the time	826 (51.7)	821 (60.4 )	1147 (57.6 )	
**Do you cover your nose with a tissue when coughing and sneezing?**				
Sometimes	90 (14.3)	149 (11.0)	239 (12.0)	0.001*
Regularly	109 (17.3)	493 (36.3)	602 (30.3)	
All the time	431 (68.4)	718 (52.8)	1149 (57.7)	

*Statistically significant at *p*<0.05

Concerning the participants’ level of trust towards COVID-19 protective measures and their commitment to practicing precautionary measures in the community, there were significant differences between males and females as shown in Table 2.

**Fig. 1:**
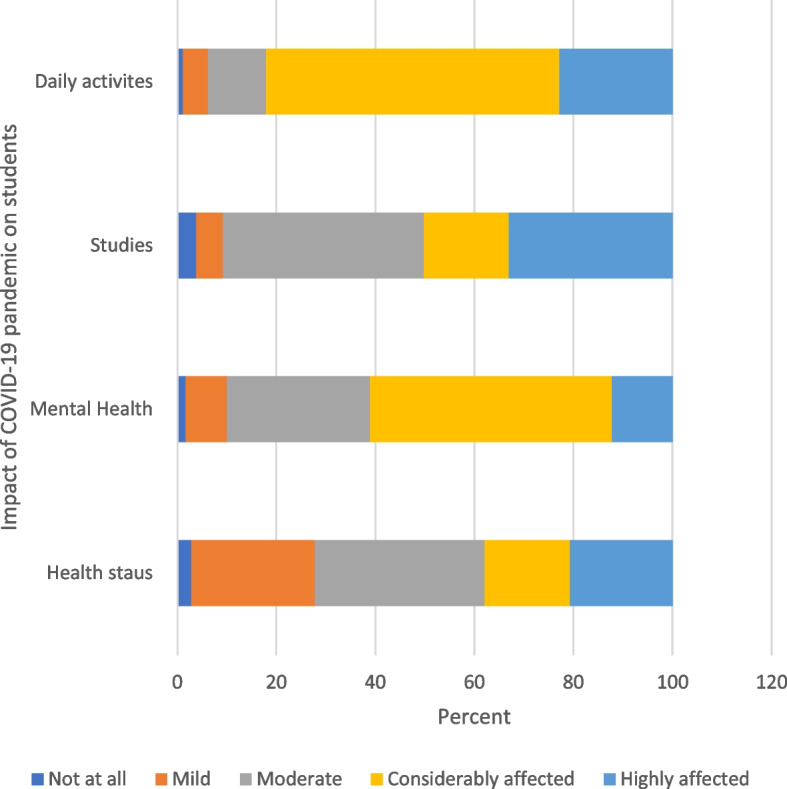
Impact of COVID-19 pandemic on students’ health and quality of life

**Fig. 2:**
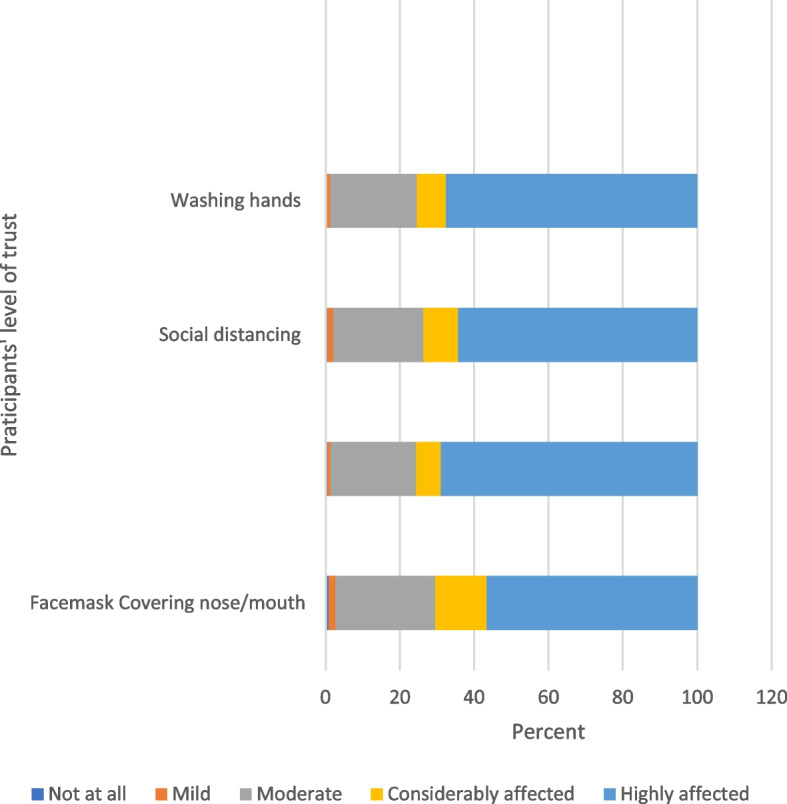
Students’ level of trust towards COVID-19 protective measures

Figures 1 and 2 illustrate the participants’ perceived impact during the pandemic and their trust level toward COVID-19 protective measures using the 5-point Likert scale.

In Table 3, attitudes and practices among students are presented in relation to their residences. Urban residents complained about mental issues and a high level of affection regarding their health status (*p*<0.001 and *p* = 0.019, respectively). In addition, the participants’ level of trust towards COVID-19 protective measures and their commitment to practicing preventive measures in the community were statistically different between urban and rural communities as shown in Table 3.

**Table 3:** Participant’s attitudes and practices toward the COVID-19 pandemic by residences (*n*=1990)

Variables	Rural	Urban	Total	***p***-value
	***N***= 835 (42.0%)	***N***=1155 (58.0 %)	***N***= 1990 (100%)	
**How disturbed have you been in the past 3 months regarding your**				
**Mental Health**				
Mild	84 (10.1)	114 (9.9)	198 (9.9)	0.001*
Moderate	253 (30.3)	323 (28.0)	576 (28.9)	
Severe	498 (59.6)	718 (62.2)	1216 (61.1)	
**Health status**				
Mild	236 (28.3)	317 (27.4)	553 (27.8)	0.019*
Moderate	309 (37.0)	372 (32.2)	681 (34.2)	
Severe	290 (34.7)	466 (40.3)	756 (38.0)	
**Studies achievement**				
Mild	60 (7.2)	123 (10.6)	183 (9.2)	0.129
Moderate	354 (42.4)	453 (39.2)	807 (40.6)	
Severe	421 (50.4)	579 (50.6)	1000 (50.3)	
**Daily activities**				
Mild	54 (6.5)	69 (6.0)	123 (6.2)	0.956
Moderate	118 (14.4)	114 (9.9)	232 (11.7)	
Severe	663 (79.4)	972 (84.2)	1635 (82.2)	
**Trusting the protective measures (Level of confidence)**				
**Wearing the mask**				
Low	14 (1.7)	38 (3.3)	52 (2.6)	
Moderate	228 (27.3)	305 (26.2)	533 (26.8)	0.030*
High	593 (71.0)	812 (70.3)	1407 (70.46)	
**Covering nose and mouth during sneezing and coughing**				
Low	4 (0.5)	22 (1.9)	26 (1.3)	0.001*
Moderate	119 (23.8)	258 (22.3)	457 (23.0)	
High	632 (75.5)	875 (75.8)	1507 (75.7 )	
**Social distancing**				
Low	14 (1.7)	27 (2.3)	41 (2.1)	0.003*
Moderate	228 (27.3)	256 (22.2)	484 (24.3)	
High	593 (71.0)	872 (75.5)	1465 (73.6)	
**Washing hands with soap and water**				
Low	6 (0.7)	18 (1.6)	24 (1.2)	0.001*
Moderate	197 (23.6)	267 (23.1)	464 (23.3)	
High	632 (75.7)	870 (75.3)	1502 (75.5)	
**Practices of precautionary actions in the community**				
**Do you use a facemask when leaving home?**				
Sometimes	96 (11.5)	161 (13.9)	257 (12.9)	0.003*
Regularly	393 (47.1)	572 (49.5)	965 (48.5)	
All the time	346 (41.4)	422 (36.5)	768 (38.6)	
**Do you avoid crowded places to protect yourself from COVID-19?**				
Sometimes	184 (22.0)	157 (13.6)	341 (17.1)	0.001*
Regularly	348 (41.7)	416 (36.0)	764 (38.4)	
All the time	303 (36.3)	582 (50.4)	885 (44.5)	
**Do you practice social distancing to protect yourself?**				
Sometimes	0 (0.0)	525 (45.5)	525 (26.4)	0.003*
Regularly	0 (0.0)	528 (50.4)	528 (29.2)	
All the time	835 (100)	48 (4.2)	883 (44.4)	
**Do you use disinfectant or hand gel to wash your hands to protect yourself?**				
Sometimes	101 (12.1)	172 (14.9)	273 (13.7)	0.001*
Regularly	256 (30.7)	314 (27.2)	570 (28.9)	
All the time	478 (57.2)	669 (57.9 )	1147 (57.6 )	
**Do you cover your nose with a tissue when coughing and sneezing?**				
Sometimes	45 (5.4)	194 (16.8)	239 (12.0)	0.001*
Regularly	306 (36.6)	296 (25.6)	602 (30.3)	
All the time	484 (58.0)	665 (57.6)	1149 (57.7)	

*Statistically significant at *p*<0.05

**Fig. 3:**
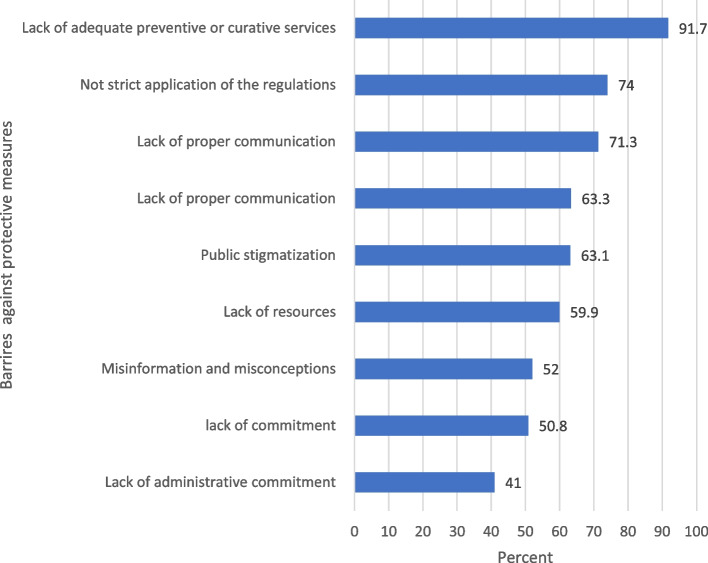
Barriers to adhere to preventive Measures for COVID-19 protective measures

Table 4 displays the mean scores of the participants’ attitudes towards the pandemic, protective measures, and practices of precautionary actions in the community. A statistically significant difference was detected regarding their level of concerns and their perception of health status (*p*<0.05). Figure 3 demonstrated public health constraints and social measures for COVID-19 protective measures among the study population.

**Table 4:** Attitudes and practices mean scores in relation to participants’ concern and perceived health status (*n*=1990)

Variables	Attitudes toward the pandemic	***F*** ^**a**^	***p***-value	Attitudes toward protective measures	***F*** ^**a**^	***p***-value	Practices toward COVID-19	***F*** ^**a**^	***p***-value
	Mean ±SD			Mean ±SD			Mean ±SD		
**Concerns toward the pandemic**									
Not concerned	13.6 ± 2.7	51.03	0.001*	20.2 ± 4.3	9.462	0.001*	11.5 ± 1.6	4.590	0.003*
Well concerned	13.9 ± 2.6			21.5 ± 3.9			11.7 ± 1.6		
Quite concerned	14.7 ± 2.5			21.5 ± 3.7			11.7 ± 1.7		
Extremely concerned	15.9 ± 2.7			21.9 ± 3.8			11.3 ± 1.7		
**Perceived health status**									
Bad/fair	14.8 ± 2.6	33.32	0.001*	20.9 ± 4.0	23.307	0.001*	12.3 ± 1.5	0.223	0.001*
Average/good	14.0 ± 2.6			22.1 ± 3.5			11.0 ± 1.6		
Very good/excellent	15.3 ± 2.7			21.2 ± 3.7			10.2 ± 1.3		

*Statistically significant at *p*<0.05

^a^ANOVA test

Table 5 illustrates that the participants’ attitude toward the pandemic was significantly positive among those who perceived a higher risk of infection, were more willing to proactively report actively confirmed cases to the authority, and would seek medical advice if experiences symptoms of COVID-19 (*p*<0.05). In addition, the attitude towards protective measures was significantly positive in all comparisons except for residence. Concerning their practices of precautionary actions, a significantly higher score was detected in students from rural areas, those who perceived themselves as at risk of infection, students willing to report their contact with confirmed cases to the authority, and those who avoided contact with the positive cases (Table 5).

**Table 5:** Attitudes and practices mean scores of study participants toward COVID-19 pandemic prevention (*n*=1990)

Variables	Attitudes toward the pandemic	***t*** ^**a**^	***p***-value	Attitudes toward protective measures	***t*** ^**a**^	***p***-value	Practices toward COVID-19	t ^**a**^	***p***-value
	Mean ± SD			Mean ± SD			Mean ± SD		
**Gender**									
Male	15.4 ± 2.8	3.743	0.436	20.6 ± 4.1	15.78	0.001*	11.5 ± 1.6	1.766	0.728
Female	14.6 ± 2.6			21.5 ± 3.6			11.6 ± 1.7		
**Residences**									
Rural	14.5 ± 2.5	4.814	0.160	21.4 ± 3.7	0.466	0.106	12.4 ± 1.4	0.223	0.001*
Urban	14.6 ± 2.7			21.1 ± 3.8			11.0 ± 1.6		
**Perceived risk of infection**									
Yes	14.8 ± 2.6			21.9 ± 3.6			12.7 ± 1.5		
No	14.3 ± 2.7	4.501	0.001*	20.8 ± 3.9	19.44	0.001*	10.8 ± 1.5	3.374	0.001*
**Contact cases**									
Reporting to the authority	14.7 ± 2.6	1.103	0.005*	21.2 ± 3.9	0.015	0.001*	11.8 ± 1.6	0.345	0.001*
Don’t know	14.3 ± 2.7			21.7 ± 3.7			10.9 ± 1.6		
**Avoid contact with positive case**									
Yes	14.6 ± 2.6			19.9 ± 4.3			11.6 ± 1.7		
No	14.2 ± 2.3	0.435	0.924	21.3 ± 3.8	3.521	0.040*	11.2 ± 1.6	0.126	0.009*
**Flu symptoms/avoid contact with others**									
Yes	14.6 ± 2.6	0.059	0.329	21.5 ± 3.7	15.01	0.001*	11.6 ± 1.7	0.301	0.107
No	14.4 ± 2.6			19.7 ± 4.3			11.8 ± 1.7		
**Experiences infection/seeking medical advise**									
Yes	15.9 ± 2.6	0.359	0.001*	22.2 ± 3.7	0.216	0.045*	11.2 ± 1.6	0.230	0.067
No	14.5 ± 2.6			21.2 ± 3.8			11.6 ± 1.8		

*Statistically significant at *p*<0.05

^a^ Independent sample *t*-test

## Discussion

The use of precautionary measures is essential to reduce the spread of the COVID-19 infection, particularly among the active youth attending educational institutions. In addition, medical, nursing, and pharmacy students represent their families and their social circle a reliable source of information about the epidemic and scientific methods to decrease the risk of infection. The present study aimed to assess the perceptions and attitudes of Egyptian university students in health sector faculties towards the COVID-19 pandemic prevention. The results of this study highlighted the gap in practice or potential factors that lead to a less-than-required attitude of university students in health sector faculties. This can help higher education stakeholders develop an educational program that addresses medical students to increase their awareness of the epidemic and the value of protective measures.

### Perception of medical and nursing students towards COVID-19 prevention

Perception of risk is essential to motivate adherence to protective measures. In the present study, most of the participants perceived the seriousness of the COVID-19 pandemic (88.2%), and this result is harmonized with a study of attitudes, anxiety, and behavioral practices regarding COVID-19 among university students in Jordan. Olaimat et al., [15] reported that 69.1% of students showed a positive attitude towards the seriousness of COVID-19. Furthermore, in a study on knowledge, attitudes, and practices of medical students regarding COVID-19 in Afghanistan, it was reported that the majority (91%) believed that COVID-19 was a serious disease [16]. However, only half of the students realized that they are at risk of infection. Almost 64.1% are quite and deeply concerned about the pandemic. This may explain why adherence to protective measures all the time was lower than anticipated in the studied sample.

Similarly, Sultan et al. [17] medical students surveyed at Suez Canal University, Egypt, reported that 83% of students agreed that COVID-19 is life-threatening, 76.3% recognized the risk of infection during ward rotations, while only 33.9% believed that being a medical student increased their susceptibility. In addition, Hussein et al. [18] surveyed medical students at Ain Shams University, Egypt, and found that approximately 92% agreed/strongly agreed that the disease is serious, but only 38.8% thought that they are at higher risk of being infected than other people.

### The impact of COVID-19 on students’ health and quality of life:

Regarding the effect of the pandemic on the students’ mental health and psychological troubles, health status, studies achievement/educational performance, and daily activities, statistically significant differences were detected between male and female students regarding their psychological distress and health status toward the pandemic in the last 3 months (*p* = 0.001 and 0.019, respectively). These results are in agreement with, the study of nursing students’ attitudes, knowledge, and willingness of to receive the coronavirus disease vaccine Aslan & Pekince [10] illustrated that the stress levels of female students were found to be higher (*P* < .001).

Also, Rivera-Lozada et al., [19] reported that the aspects most affected by the COVID-19 pandemic among healthcare professionals were mental health and daily activities with a total of 37.4% (*n* = 113) obtained scores above the 75th percentile, with a predominance of fear of becoming infected (49.7%), returning home and infecting the family (45%) and fearing of death from COVID 19 (49.7%) [20]. Having sufficient knowledge about the spread of the disease and the role of protective measures can reduce these fears and anxieties as research suggests that lack of knowledge can result in the overestimation of risks.

### Students’ attitude towards COVID-19 prevention

When assessing the attitude score towards the COVID-19 pandemic and its secondary impacts, significantly high scores were detected in those quite-to-extremely concerned, very good/excellent status, perceiving a risk of infection, willing to report their contact with positive cases & avoid contact with them, and seeking medical advice on infection. It seems that individuals with these criteria are more likely to adhere to distancing and restrict their outdoor activities except for necessity, which in turn would impact their daily life and their mental health. Strict adherence to the recommended protective measures represents the only choice for limiting the spread of the novel coronavirus. The WHO and Centers for disease control and prevention (CDC) have released published statements regarding the recommended measures that should be adopted [3, 13].

Furthermore, Salem et al., [21] in their assessment of knowledge, attitudes, and precautionary actions against COVID-19 among medical students in Egypt reported that more than eighty percent of the respondents were willing to take action to prevent the spread of misconceptions and rumors in their communities against COVID-19 and showed their willingness to volunteer to raise community awareness.

The current study analyzed the rate of trust and practice of protective measures among medical students. About 97% of students moderately/highly trusted that social distancing, wearing masks, covering nose and mouth while coughing or sneezing, and handwashing can prevent the spread of infection. This is consistent with the study by Sultan et al. [17] who found that 79% to 92% of students correctly identified the recommended preventive measures. Abd El Fatah et al. [22] reported substantially higher rates of medical students recognizing the value of the recommended protective measures, reaching over 97%.

These results are in agreement with the study of risk perception regarding the COVID-19 outbreak among the general population: a comparative Middle East survey, Shahin & Hussien, [13] revealed that slightly more than two-thirds of the study sample were most certain that frequent hand hygiene and agreed that maintaining social distancing and implementing quarantine most certainly helps prevent COVID-19 infection (73.0% and 78.4%, respectively).

Attitude toward protective measures in the present study was significantly higher in those quite-to-extremely concerned, very good/excellent status, perceiving a risk of infection, willing to report contact with positive cases and avoid contact with them, seeking medical advice on infection, having average health status, female students, and those avoiding contact when experiencing flu symptoms. Most of these differences were anticipated as students realized the magnitude of the problem or those concerned with avoiding infection would use and methods that were recommended to safeguard themselves. However, gender differences cannot be precisely identified.

### Students practices toward COVID-19 prevention

With respect to recommended protective measures practices, the majority of students showed compliance regularly or all the time (90% or above). Previous studies have reported varying patterns and rates of protective measures practices. In a study of the knowledge, attitudes, and practices of Moroccan nursing students Fakhri et al., [23] detected that almost all participants reported avoiding crowded places frequently. About 93.4% frequently wear face masks when leaving home, and 85.5% maintain social distancing frequently. However, only 47.4% reported that they frequently washed their hands. Approximately 51% stated that the coronavirus outbreak has considerably changed their daily routines. While in a selected nursing institution in Saudi Arabia, Begum, [24] reported that 77.4% of students wash their hands frequently with soap and water for at least 20 to 40 s, 91.9% follow social distancing to avoid contact of infected persons, 83.9% avoid going to crowded places these days, 75.8% practice good respiratory hygiene and avoiding touching the eyes, nose or mouth with unwashed hands and 87.1% have worn masks when going out in recent days.

Abd El Fatah et al. [22] (14) reported a higher rate of 95% for students washing their hands several times per day but wearing face masks and cleansing surfaces with disinfectants was practiced by much lower rates (38 to 45% and 82% to 83%, respectively). Sultan et al. [17] found that approximately 90% of students or above reported practicing the preventive measures, while the least practiced measure was disinfection and cleaning (88.3%). Hussein et al. [18] reported low rates of avoiding crowded activities and societies (61.8%), disinfecting frequently contacted hard objects like door handles (52.4%), and discussing preventive measures with the family and friends (60.4%). Other behaviors were performed by a higher percentage as decreasing the use of public transportation (87.5%), using tissues while sneezing/coughing (75.8%), and washing hands frequently (73.8%).

Elsayed Emara et al. [25] surveyed students of the Faculty of Medicine in Tanta, Egypt, and found that the majority of students used disinfectants and avoided crowded places (95% and 76.8%, respectively) while wearing face masks when leaving home was practiced by only 56.7% of the participants. An Iranian study reported rates of about 94% of practicing the protective measures, while disinfection and cleaning were reported by 85.6% [26]. Whereas, in a study on awareness and knowledge of COVID-19 among senior pharmacy students in Egypt Hamza et al., [27] reported that 87% of students answered that they did not go out to any crowded place but approximately 50% of students admitted that they did not wear masks when they left their house.

Discrepancies in the reported rates and more practiced measures among the studies may be explained by the variations in chronological order which may affect the knowledge prevalent at the time, and by variations in the regions from which the students were recruited. In addition, some studies included only medical students who are expected to have higher knowledge and practice levels, while other studies, such as the present study, included students of pharmacy and nursing colleges. Moreover, the difference in practice can be impacted by the academic level of the student, as final year students must demonstrate higher knowledge and familiarity with sanitary habits.

### Barriers to compliance with preventive measures

With regard to the current study, it is evident that the rates of those practicing the measures all the time were mostly 50% or less, the least of which was wearing face masks on leaving home. This indicates that some barriers may hinder the application of the measures by the students in all situations. The most frequently mentioned barriers against compliance with the protective measures were the lack of preventive/curative services, the absence of strict application of regulations, poor communication, public stigmatization, and the lack of resources. There was a shortage in face masks and sanitizers during the first few months after the declaration of the pandemic, but now they are available at reasonable prices. However, the cost still represents a burden on the budget of students who have to attend daily in their colleges.

While in a study on the predictors of compliance with standard precautions among nursing students in China, Rn et al., [28] demonstrated that the perceived barriers included difficulty in performing procedures properly when wearing personal proactive equipment (39.3), offending patients when wearing personal proactive equipment to provide care (28.7%), too busy to follow standard precautions (SPs) (23.9%), and abusive nursing staff-ward practice if NSs followed proper SPs (22.7%).

Another barrier to complying with the protective measures in the current study is the social stigma arises from the misconception of laypeople that only the persons infected with COVID-19 are wearing masks. Furthermore, a study on community risk perception and barriers to practicing COVID-19 prevention measures in Northwest Ethiopia conveyed that the major identified barriers for practicing COVID-19 prevention measures were the presence of strong cultural and religious practices, perceiving that the disease does not affect the young, misinformation about the disease, and lack of trust in prevention measures [29]. Overcoming these barriers can increase the rate of compliance with the measures, which in turn will slow the spread of infection and reduce the number of new cases.

### Factors affecting student’s adherence to protective measures

Some factors that may affect adherence to protective measures were assessed in the present study. Significantly higher practice scores were detected in those with poor health status, rural areas, perceiving the risk of infection, willing to report their contact with positive cases, and avoiding contact with positive cases.

Some practices of protective measures were significantly more prevalent in one gender or the other. A high percentage of female students were adherent regularly/all the time to wear a facemask, social distancing, and using disinfectant or hand gel, while a larger percentage of male students were avoiding crowded places and covering the nose with a tissue when coughing or sneezing. However, the total score did not differ significantly between the two genders. This is in line with the findings of Hussein et al. [18] who found no statistically significant differences in the total performance score between male and female subjects. Meanwhile, Salem et al. [21] stated that only handwashing with soap and water was significantly more frequent among female students. Hamza et al. [27] also reported that females preferred to maintain social distance more than male students (OR 2.3, 95% confidence interval [CI] 1.2–4.4). Elsayed Emara et al. [25] found no significant difference between male and female students in wearing face masks, using disinfectants, and avoiding crowded places., Sultan et al. [17] found a significantly higher degree of preventive measures among female students.

These findings of higher practice scores among those who are aware of the risk of infection since a high level of risk perception can motivate individuals to strictly practice the protective measures. Also, reporting of their contact with positive cases to the concerned authorities and avoiding contact with them represents behaviors that reflect the perception of risk and an intention to protect oneself from infection, and both behaviors showed high practice scores. Sultan et al. [17] and Hussein et al. [18] similarly detected a significantly higher performance score in those with high-risk perception levels.

Interestingly, we found that the rates of wearing facemask outdoors, social distancing, using disinfectants, and covering nose and mouth while sneezing/coughing regularly/all the time were significantly higher among students from rural areas, while the rate of avoiding crowded places was higher among urban students. In contrast to our findings, Salem et al. [21] reported a significantly higher practice score among students from urban areas.

These variations in practicing the protective measures among urban and rural regions can be attributed to differences in the included universities across the studies. The effect of society and its culture on the practice of protective measures may differ even among urban communities depending on the degree of openness and culture of the community. Some urban districts may be less educated while some rural regions may have a highly educated population. These factors are difficult to be determined in an online questionnaire, but may provide a potential explanation of these discrepancies across studies from the same country.

The attitude of Egyptian medical students during the COVID-19 pandemic has been assessed by some previous studies; nonetheless, the present study has several strengths besides covering some knowledge gaps that were not elucidated before. The study included students from all health sector-related colleges (colleges of medicine, pharmacy, and nursing), not only the students of the faculty of medicine. Students were recruited from more than one university, which resulted in a sample that represented the different cultural and geographic variations that can contribute to the students’ attitudes and practices.

The extent to which protective measures are applied was investigated and the underlying causes beneath the non-compliance with the measures were explored, forming the basis for designing the intervention programs that should address the problem. However, the present study was subject to few limitations. The effect of the academic level on attitude and practices has not been investigated. Moreover, the definition of rural and urban areas was left to the students’ choice in the questionnaire, and this may have resulted in some misclassifications. Another more precise classification of the residence is warranted, probably according to the socioeconomic status as well as the geographic location.

## Conclusion

The adherence of university students from health sector faculties to the protective measures should be encouraged and the effectiveness of preventive measures should be stressed to enhance the positive practices of medical students. Designing educational programs is required to inform and train university students from health sector faculties about their expected role in combating the pandemic.
